# Measured and Simulated Nitrous Oxide Emissions from Ryegrass- and Ryegrass/White Clover-Based Grasslands in a Moist Temperate Climate

**DOI:** 10.1371/journal.pone.0026176

**Published:** 2011-10-10

**Authors:** Dejun Li, Gary Lanigan, James Humphreys

**Affiliations:** 1 Livestock Systems Research Department, Teagasc, Moorepark, Fermoy, County Cork, Ireland; 2 Biosystem Engineering, UCD School of Agriculture, Food Science and Veterinary Medicine, University College Dublin, Belfield, Dublin, Ireland; 3 Environment, Soils and Land-Use Research Department, Teagasc, Johnstown Castle, County Wexford, Ireland; Argonne National Laboratory, United States of America

## Abstract

There is uncertainty about the potential reduction of soil nitrous oxide (N_2_O) emission when fertilizer nitrogen (FN) is partially or completely replaced by biological N fixation (BNF) in temperate grassland. The objectives of this study were to 1) investigate the changes in N_2_O emissions when BNF is used to replace FN in permanent grassland, and 2) evaluate the applicability of the process-based model DNDC to simulate N_2_O emissions from Irish grasslands. Three grazing treatments were: (i) ryegrass (*Lolium perenne*) grasslands receiving 226 kg FN ha^−1^ yr^−1^ (GG+FN), (ii) ryegrass/white clover (*Trifolium repens*) grasslands receiving 58 kg FN ha^−1^ yr^−1^ (GWC+FN) applied in spring, and (iii) ryegrass/white clover grasslands receiving no FN (GWC-FN). Two background treatments, un-grazed swards with ryegrass only (G–B) or ryegrass/white clover (WC–B), did not receive slurry or FN and the herbage was harvested by mowing. There was no significant difference in annual N_2_O emissions between G–B (2.38±0.12 kg N ha^−1^ yr^−1^ (mean±SE)) and WC-B (2.45±0.85 kg N ha^−1^ yr^−1^), indicating that N_2_O emission due to BNF itself and clover residual decomposition from permanent ryegrass/clover grassland was negligible. N_2_O emissions were 7.82±1.67, 6.35±1.14 and 6.54±1.70 kg N ha^−1^ yr^−1^, respectively, from GG+FN, GWC+FN and GWC-FN. N_2_O fluxes simulated by DNDC agreed well with the measured values with significant correlation between simulated and measured daily fluxes for the three grazing treatments, but the simulation did not agree very well for the background treatments. DNDC overestimated annual emission by 61% for GG+FN, and underestimated by 45% for GWC-FN, but simulated very well for GWC+FN. Both the measured and simulated results supported that there was a clear reduction of N_2_O emissions when FN was replaced by BNF.

## Introduction

Nitrous oxide (N_2_O) is a potent greenhouse gas (GHG) with a global warming potential 298 times higher than carbon dioxide over a 100-year time horizon [1]. In 2008, it contributed about 6.2% to the overall global radiative forcing [2]. In addition, N_2_O currently is the single most important stratospheric ozone-depleting substance and is expected to remain the largest throughout the 21st century [3]. Globally averaged mixing ratio of N_2_O reached 321.8 ppb in 2008 with a mean annual increase of 0.78 ppb over the past 10 years [2]. Accordingly, the measurement and abatement of N_2_O emissions are imperative.

Agricultural soils are the major source of N_2_O, responsible for about 35% of annual global emissions [4]. However, there are significant uncertainties in the inventory estimate of N_2_O emissions from agricultural soils with a range from 0.6 to 14.8Tg N yr^−1^ (1 Tg = 10^12^ g) despite many years' measurements [5]. N_2_O in soils is naturally produced by nitrification, denitrification and other processes like nitrifier denitrification [6], and is often enhanced where available N exceeds plant requirements, especially under wet conditions [7]. Agricultural activities have significantly enhanced N_2_O emissions by increasing available N in soils through application of fertilizer N (FN) and manures. For example, in the European Union (EU-15), 40% of the direct soil emission is attributed to FN application, and another 21% to manure application [8]. The global use of FN has increased sevenfold between 1960 and 1995 and is expected to increase another threefold by 2050 unless there is a substantial increase in FN use efficiency [9]. Based on current trends in FN use, N_2_O emissions from agricultural soils were projected to increase by 47% in 2020 relative to 1990 [10]. There is necessity to explore strategies that will sustain agricultural production while lower soil N_2_O emissions by reducing the use of FN.

Globally, grassland-based agriculture is the major part in agriculture sector. Of all the agricultural land, 68% is permanent pastures [11]. Incorporation of N-fixing legume species provides a potential to lower N_2_O emission from grassland by partly or completely replacement of FN by biological N fixation (BNF). White clover is the main legume in pastures and meadows of temperate regions [12]. Most often, white clover is grown with companion grasses. This fixed N becomes available slowly over time to the companion grass after it is released into soil *via* exudates from living legume roots, by mineralization of senesced legume tissues and in excreta after consumption by grazing animals [13]. Davies and Hopkins [14] reported that, under simulated grazing (frequent mechanical harvests and no returns of excreta) herbage production from clover-based grassland was similar to that from perennial ryegrass receiving fertilizer N input of 100 to 200 kg ha^−1^. From system-scale dairy production experiments, Humphreys et al. [15] concluded productivity of clover-based systems receiving FN input of 90 kg ha^−1^ in spring was similar to perennial ryegrass receiving FN input of 226 kg ha^−1^ or approximately 80% of perennial ryegrass receiving FN inputs of 350 to 413 kg N ha^−1^. There is evidence that N use efficiency in clover-based systems is higher than FN based systems [16], [17]. This implies that N_2_O emission in the clover-based grassland may be lower since N use efficiency is negatively related to N_2_O emissions [18], [19].

Although the use of white clover to replace FN inputs was proposed as an option for reducing N_2_O emissions from grassland [20], [21], there are a very limited number of studies comparing N_2_O emissions from fertilizer- and white clover-based systems under similar conditions. Isotope tracing studies have estimated direct N_2_O emissions from clover only accounted for 2.1±0.5% of the total N_2_O emission from white clover-based systems [22]. In addition, more recent studies have found lower N_2_O emissions on ryegrass/clover pastures than on ryegrass monocultures after 80kg N was applied [23]. This is probably due to higher N utilization in the mixtures [17]. In a recent IPCC (the Intergovernmental Panel on Climate Change) report, use of forage legumes has been proposed as a measure to lower greenhouse gas emissions from grassland, but there is uncertainty whether this measure can lower soil N_2_O emissions [7]. Based on a comprehensive literature survey, Rochette & Janzen [24] suggested that evidence to date for direct release of N_2_O from BNF itself was inadequate to justify the universal adoption of an emission factor similar to that of Fertilizer N (1.25%) in global inventories of N_2_O emission, and stressed that further research was merited.

In this study, we compared N_2_O emissions from both ryegrass-based and ryegrass/white clover based grasslands in Ireland. Field measurements were compared with results simulated with a process-base model DNDC (denitrification-decomposition) [25], [26]. We hypothesized that at the same level of productivity N_2_O emissions from ryegrass-based grasslands, which usually receive high levels of fertilizer N, in Ireland would be larger than those from ryegrass/white clover-based grasslands. Specific objectives were to 1) evaluate the contribution of BNF to N_2_O emission in permanent grassland, 2) investigate the potential of white clover to lower soil N_2_O emissions from typical dairy production systems in Ireland, 3) determine annual rates of N_2_O emission from the studied grasslands, and 4) assess the applicability of DNDC model to predict N_2_O emissions from typical grassland systems in Ireland.

## Materials and Methods

### Site description

The study was conducted at the Teagasc Solohead Research Farm (52°51'N, 08°21'W). This site is located on a flat to gently undulating land with an altitude of approximately 79 m above sea level. This region has a temperate maritime climate with the mean annual rainfall and soil temperature (1998–2008) of 991 mm and 11.1°C, respectively. The mean monthly minimum temperature varied between 4.3 and 7.8°C in December, January or February, and the mean monthly maximum soil temperature ranged between 15.6 and 18.2°C occurring in July or August.

The soil, classified as poorly draining clay loam, has sand, silt and clay contents of approximately 34%, 36%, 29%, respectively, in the surface layer (0–10 cm). The soil bulk density at 0–5 cm depth is 0.86 g cm^−3^. Soil pH (H_2_O), cation exchange capacity, total N and total C content in the surface soil were 6.5, 0.3 meq g^−1^, 0.54% and 5.35%, respectively. More detailed description of the farm management and production are presented elsewhere [15], [27].

### Experimental design

The experiment was a randomized block design with five treatments and three replicates (Table 1). The treatments were: 1) grazed perennial ryegrass (*Lolium perenne*) swards receiving high rate of FN (GG+FN), 2) grazed ryegrass/white clover (*Trifolium repens*) swards receiving low rate of FN (GWC+FN), 3) grazed ryegrass/white clover swards not receiving FN (GWC-FN), 4) perennial ryegrass plots (G–B) and 5) perennial ryegrass/white clover plots (WC-B). The swards (paddocks) of treatments GG+FN, GWC+FN and GWC-FN were rotationally grazed by dairy cows and have under the same treatment since the beginning of 2003 (GG+FN and GWC+FN) or 2008 (GWC-FN). The area of theses paddocks ranged from 0.32 to 1.63 ha. G-B and WC-B, which were used to measure the background N_2_O emissions (N_2_O_Bk_), which is defined as soil N_2_O emission from unfertilized and mown-only grassland [28], from perennial ryegrass and ryegrass/white clover swards, respectively, were not grazed and did not receive slurry or FN. The area of each plot was 10 m×10 m. Herbage in these plots was mown at the beginning of each grazing event to 5 cm height to coincide with each grazing event on the corresponding grazed paddocks, with clippings removed. All five treatments were imposed from February 2008 and N_2_O measurements began in October 2009. The application dates and amounts of slurry and FN are shown in Table 1. For GWC+FN and GWC-FN, there is additional N input besides slurry or FN due to biological N fixation which was estimated to be 87.5 and 116.5 kg N ha^−1^ yr^−1^ based on measurement in 2008 and 2009 [29].

**Table 1:** Grazing and application of slurry and fertilizer N in 2009 and 2010.

	GG+FN	GWC+FN	GWC-FN	G-B	WC-B
Grazing	+^a^	+	+	-	-
Slurry (m^3^ ha^−1^) and fertilizer N (kg N ha^−1^) application in 2009					
Slurry (9 Feb)^b^	34^c^	34	34	-	-
Urea (25 Mar)	28.4	28.4	-	-	-
CAN (8 May)	51	34	-	-	-
CAN (23 Jun)	26	-	-	-	-
CAN (14 Jul)	26	-	-	-	-
CAN (4 Aug)	26	-	-	-	-
CAN (1 Sep)	26	-	-	-	-
Slurry (m^3^ ha^−1^) and fertilizer N (kg N ha^−1^) application in 2010					
Slurry (10 Feb)	28	28	28	-	-
Urea (14 Apr)	57.5	57.5	-	-	-
CAN (30 Jun)	33.8	-	-	-	-
CAN (22 Jul)	67.5	-	-	-	-
CAN (16 Aug)	67.5	-	-	-	-

Note: ^a^ + and – denote with and without, respectively; ^b^ the dates in the parentheses mean when slurry, urea and CAN (calcium ammonium nitrate) were applied.

Pasture was allocated to cows in a rotational grazing system and post-grazing heights, measured with a rising plate meter (Grasstec, Charleville, Ireland), were used to determine when cows moved to the next section. Post-grazing heights were maintained at approximately 50 mm throughout the grazing season. Rotations were approximately 24 d in length during the main grazing season between April and September. Cow numbers per paddock were managed to maintain the same rotation lengths on each treatments and the average stocking densities were 2.2 cows ha^−1^ for both GG+FN and GWC+FN, but the stocking density for GWC-FN was 1.6 cows ha^−1^. A study conducted during 2004 to 2006 indicated that with the same stocking density (2.2 cows ha^−1^) there was no statistical difference in milk production between GG+FN and GWC+FN (14.3 ton ha^−1^ yr^−1^ for both systems) with concentrate supply of 541 and 559 kg DM cow^−1^ for GG+FN and GWC+FN, respectively [15]. Another study conducted in 2008/2009 revealed that milk production was much higher for GWC+FN (13.7 ton ha^−1^ yr^−1^) than for GWC-FN (10.4 ton ha^−1^ yr^−1^) [30].

### N_2_O flux measurements

N_2_O fluxes were measured by a static chamber technique [31]. The chambers were made of polyvinylchloride (PVC) pipe with an internal diameter of 29.5 cm and included two parts: (i) permanent collar and (ii) chamber. The collars were permanently installed in the field to a depth of 12 cm. The headspace height of the chamber was 40 cm and hence the headspace volume was 27.3 L. Inside the chamber, a thermo-sensor and a fan were installed to measure the air temperature and to ensure that air in the chamber was mixed well during sampling, respectively. Each chamber was fixed with a 3-way stopcock (Discofix). One port of the stopcock was connected with a plastic tube (10 cm long with internal diameter of 0.5 cm) entering the inside of the chamber and one port was coupled with a needle. The third port was left open when putting the chamber onto the collar to balance the pressure inside and outside the chamber. But immediately after the chamber was put onto the collar, a syringe was connected to the third port and a 10 ml gas sample (0 min) was withdrawn from inside the chamber. A second gas sample was taken after 30 min. All gas samples were transferred into pre-evacuated 7 ml screw-cap glass septum vials (Perbio Science, UK). There were three chambers for each paddock, but only one for each control plot. In total, there were nine chambers for each grazing treatment and three chambers for each background treatment.

Sampling was conducted weekly (biweekly occasionally) with an increased frequency following fertilization. In total, 42 sampling dates were covered during the period from October 2009 to September 2010. On each sampling day, flux measurement was conducted during the period of 9:00 to 12:30. Gas samples were analyzed using a gas chromatograph (GC) (Varian GC 450; The Netherlands) fitted with an electron capture detector (ECD) and automatic sampler (Combi-PAL autosampler; CTC Analytics, Zwingen, Switzerland). N_2_O concentrations at 0 min and 30 min were used to estimate N_2_O flux (g N ha^−1^ d^−1^) for each chamber assuming that N_2_O concentrations within the chamber increased linearly within the 30 min-interval. This was supported by a companion study in which N_2_O emissions from urine treated plots (to simulate urine deposition) were measured and the same chamber were used but with gas sampling collected at 0, 10, 20 and 30 min, respectively, in order to derive a N_2_O flux. The results indicated that even at the peak fluxes, N_2_O concentrations within the chamber were found to increase linearly within 30 min (unpublished data).

The precision of GC analysis, expressed as a coefficient of variation for 10 replicate injections of a low concentration standard (330 ppb for N_2_O) and a high concentration standard (10 ppm for N_2_O) was <2%. All field measurements were within the linear range of the detector. The minimum detectable concentration change was ±7 ppb for N_2_O. The detection limit of N_2_O flux was between 3.1 and 3.5 µg N m^−2^ h^−1^ (or 0.7–0.8 g N ha^−1^ d^−1^) with the chamber temperature ranges of 0–30°C. When N_2_O concentrations were below the detection limits, these fluxes were considered to be no different from zero. Gas samples were stored <48 h before analysis and tests showed no change in gas concentration during storage. A positive flux was defined as net emission to the atmosphere (source) and a negative flux was defined as consumption (sink) by the soil microbial community.

### Ancillary measurements

Daily rainfall and soil temperature (0–5 cm in depth) were recorded by a meteorological station (Campbell Scientific, Inc.) on the farm. Daily air temperature was recorded at a meteorological station about 20 km away. Soil volumetric water content (VWC) at 5 cm depth was measured using a HH2 moisture meter coupled with a Theta probe (Delta-T Devices, Cambridge, England) along with N_2_O flux measurements on some dates. Soil volumetric water contents were converted to percentage water filled pore space (WFPS) by using the soil bulk density values and soil particle density (2.65 g cm^−3^) [32].

### DNDC modeling

In this study, the Denitrification-Decomposition (DNDC, version 9.3) model was adopted. DNDC is a process-oriented computer simulation model of carbon and nitrogen biogeochemistry in agroecosystems [25], [26]. The model consists of two components. The first component, consisting of the soil climate, crop growth and decomposition sub-models, predicts soil temperature, moisture, pH, redox potential (Eh) and substrate concentration profiles driven by ecological drivers (e.g., climate, soil, vegetation and anthropogenic activity). The second component, consisting of the nitrification, denitrification and fermentation sub-models, predicts emissions of carbon dioxide (CO_2_), methane (CH_4_), ammonia (NH_3_), nitric oxide (NO), nitrous oxide (N_2_O) and dinitrogen (N_2_) from the plant-soil systems. Classical laws of physics, chemistry and biology, as well as empirical equations generated from laboratory studies, have been incorporated in the model to parameterize each specific geochemical or biochemical reaction. The entire model forms a bridge between the C and N biogeochemical cycles and the primary ecological drivers. During the past two decades, DNDC has been widely used to simulate the emissions of the above trace gases from agroecosystems including grazed grasslands [33].

In the present study, site specific data were used for modeling, including climate data (daily minimum and maximum temperature, rainfall, N concentration in rainfall), soil properties (bulk density, pH, water filled pore space (WFPS) at field capacity and wilting point, clay fraction, hydro-conductivity, soil organic carbon, soil ammonium and nitrate concentration), crop management (including slurry and fertilizer N application, grazing). WFPS at field capacity and wilting point, and hydro-conductivity were calculated using SPAW Hydrology based on the measured soil properties [34]. One possible uncertainty was that it was ammonium nitrate, not calcium ammonium nitrate that was included as a default fertilizer in the DNDC model. In this study, we chose ammonium nitrate to replace calcium ammonium nitrate when conducting modeling since it was normally operated in previous studies using DNDC [35]–[37].

Relative bias (RB) is used as a direct measure of the tendency for over or under prediction (positive or negative values, respectively). RB was calculated using the following equation [38]: 
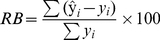
(1)


where y_i_ represents simulated values and y_i_ measured values.

### Data analysis

Since the three paddocks of each treatment were under rotational grazing, daily means of N_2_O fluxes were calculated arithmetically for each paddock. Daily means of fluxes for each treatment were the arithmetical average of three replications. Monthly, annual N_2_O emissions or emissions during grazing/nongrazing period for each paddock/plot were calculated by linear interpolation between measured daily fluxes. In order to determine the spatial variation of fluxes, daily and annual N_2_O emissions for each chamber were calculated in the same way described above. However, unless otherwise stated the reported mean values of N_2_O emission for each treatment was the arithmetical average of three replications.

ANOVA analyses with post hoc LSD tests were performed using PASW Statistics 18 (SPSS Ltd., USA) to identify differences between treatments. Difference with *P* value<0.05 was considered significant.

## Results

### Field measurements

**Figure 1:**
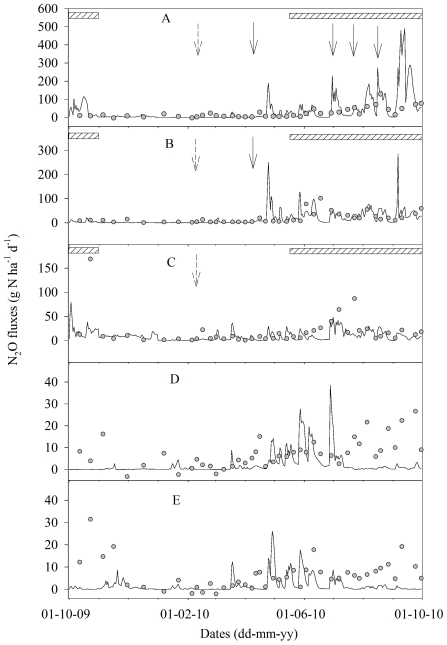
Daily N_2_O fluxes. Simulated (—) and measured (•) N_2_O fluxes for (A) GG+FN, (B) GWC+FN, (C) GWC-FN, (D) G-B and (E) WC-B. Each value is the mean of fluxes from the three paddocks or plots. Dashed or solid arrows indicate when slurry or FN was applied, respectively. The vertical edges of the shaded boxes denote the start and end of grazing period.

Daily N_2_O fluxes are presented in Figure 1. Large variation was found for all the treatments especially for the three grazing treatments. Over the sampling period, fluxes ranged between −2.7 and 129.0 g N ha^−1^ d^−1^ for GG+FN, 0.8 and 101.7 g N ha^−1^ d^−1^ for GWC+FN, 0.8 and 168.9 g N ha^−1^ d^−1^ for GWC-FN, -6.9 and 26.6 g N ha^−1^ d^−1^ for G–B and −2.1 and 31.5 g N ha^−1^ d^−1^ for WC-B. Small peaks were found following slurry application in February for GWC+FN and GWC-FN. In April, there were small peaks following urea application for both GG+FN and GWC+FN. However, substantially higher peaks were frequently encountered during the grazing period. The largest peak was found in August for GG+FN, in June for GWC+FN and in October for GWC-FN. The lowest fluxes were generally found between November and March.

For the three grazing treatments, the averaged N_2_O fluxes during the main grazing period (May to October) were 35.3 g N ha^−1^ d^−1^ for GG+FN, 28.6 g N ha^−1^ d^−1^ for GWC+FN and 33.4 g N ha^−1^ d^−1^ for GWC-FN, while those during the non-grazing period were 8.2 g N ha^−1^ d^−1^ for GG+FN, 5.3 g N ha^−1^ d^−1^ for GWC+FN and 5.1 g N ha^−1^ d^−1^ for GWC-FN, with the fluxes in the grazing period significantly higher than in the non-grazing period. However, N_2_O fluxes during the main grazing period (10.3 g N ha^−1^ d^−1^ for G-B and 10.2 g N ha^−1^ d^−1^ for WC-B) were also higher than during the non-grazing period (2.6 g N ha^−1^ d^−1^ for G-B and 3.2 g N ha^−1^ d^−1^ for WC-B).

Since the collars were kept in place throughout the sampling period, data for the same treatment can be used to assess the spatial variation of N_2_O emissions. For GG+FN, annual N_2_O emissions extrapolated based on each collar ranged from 3.38 to 18.67 kg N ha^−1^ yr^−1^, with a coefficient of variation (c.v.) of 66.9%; for GWC+FN, from 2.83 to 14.68 kg N ha^−1^ yr^−1^, with a c.v. of 57.6%; for GWC-FN, from 2.64 to 15.73 kg N ha^−1^ yr^−1^, with a c.v. of 64.4%. Considerable variation was also measured for G-B (with a c.v. of 11.2%) and WC-B (with a c.v. of 56.1%).

**Figure 2:**
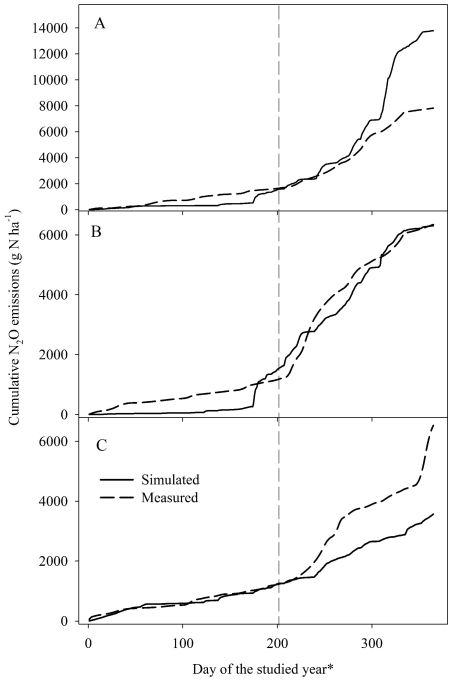
Cumulative N_2_O emissions. Cumulative N_2_O emissions over the studied year for (A) GG+FN, (B) GWC+FN, and (C) GWC-FN. The vertical broken line divided the studied year into non-grazing period (left) and grazing period (right). *The studied year composed of November-December 2009, January-September 2010 and October 2009.

Annual N_2_O emissions from WC-B (2.45±0.85 kg N ha^−1^ yr^−1^) were the same as those from G-B (2.38±0.12 kg N ha^−1^ yr^−1^) (*P*>0.05). These emissions can be regarded as background N_2_O emission (N_2_O_Bk_). Annual N_2_O emissions were 7.82±1.67, 6.35±1.14 and 6.54±1.70 kg N ha^−1^ yr^−1^ for GG+FN, GWC+FN and GWC-FN, respectively, significantly greater than N_2_O_Bk_ (*P*<0.05). No significant (*P*>0.05) differences in the annual N_2_O emissions were found among the three grazing treatments due to the large variability. However, there was an obvious trend of lower N_2_O emissions from GWC+FN and GWC-FN, where annual N_2_O emissions were 19% and 16%, respectively, lower relative to GG+FN. There was no statistical difference between GWC+FN and GWC-FN. It should be noted that due to the huge spatial and temporal variations of N_2_O emissions, large uncertainties existed in the annual estimates. For example, the large peak in October 2009 accounted for about one third (2.1 kg N ha^−1^) of the annual emission for GWC-FN, but the total emission in October 2009 was about 0.3 kg N ha^−1^ for either GG+FN or GWC+FN. In fact, most emissions were found in the grazing period for the three grazing treatments (Figure 2). For GG+FN, the non-grazing and grazing period accounted for 21.5% and 78.5% of the annual emission, respectively. The corresponding values were 19.0% and 81.0% for GWC+FN, and 19.2% and 80.8% for GWC-FN.

**Figure 3:**
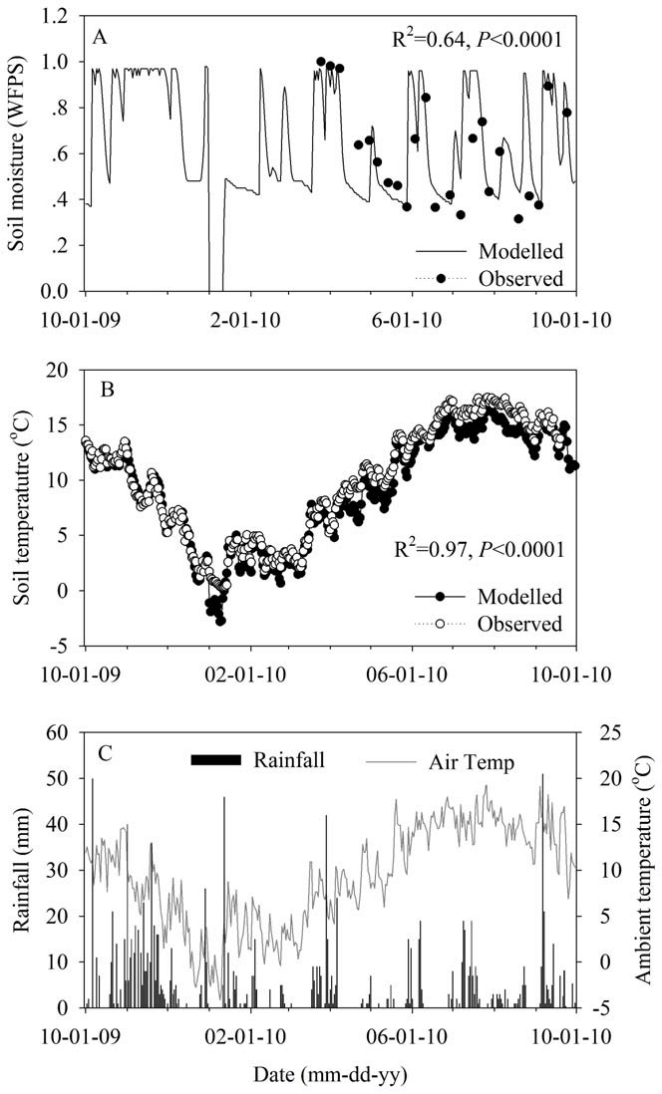
Soil microclimatic and weather conditions. (A) Simulated and measured soil water filled pore space (WFPS) at 5 cm depth, (B) simulated and measured soil temperature at 5 cm depth, and (C) daily rainfall and air temperature. Note: only measured soil moisture and temperature for GG+FN paddocks are presented here as an example.

Soil moisture and temperature at 5 cm depth were presented in Figure 3. There was no significant correlation between N_2_O fluxes and soil moisture. Strong correlation (*P*<0.05) was found between monthly N_2_O fluxes and soil temperature except GWC-FN. But there was significant relationship between monthly N_2_O fluxes and soil temperature when data in October were excluded for GWC-FN (R^2^ = 0.52, *P*<0.05).

### DNDC modeling

DNDC simulates daily plant growth with an annual herbage production of 9389, 9285 and 7814 kg DM (dry matter) ha^−1^ yr^−1^ for GG+FN, GWC+FN and GWC-FN, respectively, for year 2009. Daily growth was not measured in the present study, so the comparison between simulated and measured daily growth was not possible. The modeled and measured soil moisture and temperature along with rainfall and air temperature are presented in Figure 3. Both simulated soil moisture (*R^2^* = 0.64, *P*<0.0001, n = 23) and temperature (*R^2^* = 0.97, *P*<0.0001, n = 365) significantly correlated with the measured values.

For the three grazing treatments, DNDC simulated N_2_O fluxes quite well in comparison with the measured fluxes during the non-grazing period for GG+FN (*R^2^* = 0.76, *P*<0.001, n = 20), GWC+FN (*R^2^* = 0.82, *P*<0.001, n = 20) and GWC-FN (*R^2^* = 0.72, *P*<0.05, n = 20)(Figure 1). Although there were some discrepancies, significant correlation were found between the simulated and measured daily fluxes for GG+FN (*R^2^* = 0.27, *P*<0.001, n = 42), GWC+FN (*R^2^* = 0.11, *P*<0.05, n = 42) and GWC-FN (*R^2^* = 0.12, *P*<0.05, n = 42). The relative bias (RB) was 34.8% for GG+FN, −1.32% for GWC+FN and −42.35% for GWC-FN. However, for the two background treatments, there was no significant correlation between the simulated and measured fluxes with RB being −52.63% for G-B and −60.03% for WC-B.

It was evident that the discrepancies mainly occurred during the grazing period for the grazing treatments, i.e., in October 2009 and September 2010 for GG+FN, in May-June 2010 for GWC+FN, and in October 2009 and June-July 2010 for GWC-FN. But there was big difference between simulated and measured values in April for GWC+FN. These patterns were confirmed from the comparison of simulated and measured cumulative emissions (Figure 2), which showed that the main discrepancy occurred during the grazing period for GG+FN and GWC-FN, but there was discrepancy during the non-grazing period for GG+FN and GWC+FN, especially for the latter. DNDC simulated very well during the period from November 2009 to June 2010 for G-B (*R^2^* = 0.84, *P*<0.01, n = 8), and from December 2009 to May 2010 for WC-B (*R^2^* = 0.89, *P*<0.01, n = 6).

The simulated annual N_2_O emissions were 13.79, 6.31 and 3.57 kg N ha^−1^ yr^−1^ for GG+FN and GWC+FN and GWC-FN. The simulated annual N_2_O emission was 76% higher than the measured emission for GG+FN, and annual emission was underestimated by 45% for GWC-FN. There was little difference between the simulated and measured annual emission for GWC+FN. For G-B and WC-B, the simulated annual emissions were 0.81 and 0.65 kg N ha^-1^ yr^−1^, respectively, with RB of −66% and -74% relative to the measured values.

## Discussion

### Temporal and spatial patterns of N_2_O emissions

Distinct seasonal patterns of N_2_O emissions from grazed grasslands have been observed and related to fertilizer N application, excreta deposition and weather conditions [28]. Urine N deposition was the main contributor to N_2_O emissions for the intensively grazed paddocks and N_2_O emissions were found to be higher in the grazing period [28], [39], [40]. This was reconfirmed by the current study. For example, fluxes as high as 1011.2 g N ha^−1^ d^−1^ were observed from individual chambers during the grazing period. These values were comparable to the peak fluxes (800–2000 g N ha^−1^ d^−1^) observed in a study to simulate urine deposition which was conducted in the adjacent paddocks with 14.6 l urine m^−2^ (unpublished data). Therefore, the observed high fluxes during the grazing period were mainly caused by excreta deposition.

However, the distinct seasonal pattern found in G-B and WC-B, i.e., N_2_O fluxes during the main grazing period were also higher than during the non-grazing period, implied that weather conditions played an important role, and weather conditions might be a factor leading to higher emissions in the main grazing period for the other treatments. Air temperature and rainfall, which control the variation of soil temperature and soil moisture, are two main regulators of soil N_2_O production and emissions, since soil temperature and moisture control rates of nitrification and denitrification, and affect C and N mineralization, N uptake by plants, groundwater level and gas diffusion in soils, all of which regulate N_2_O flux [41]. ‘Pulsing’ N_2_O emissions were frequently observed shortly following rainfall after an extended dry period [42]. This was probably because microbial activity was low during prolonged soil dryness resulting in an accumulation of NH_4_^+^, NO_2_^−^ and NO_3_^−^ in thin water films of microsites and upon soil wetting, soil microbes can quickly use these pools, and produce pulses of N_2_O and other N gases [43]. However, in the current study none of the observed flux peaks were likely caused by ‘pulsing emission’ since there was rain events within 5 days before the peak fluxes were observed. For example, the highest peak flux in the current study was found in 23 October, but there were rains (35 mm in total) every day during 19–22 October (Figure 1 and Figure 3). This further confirmed that the observed flux peaks were caused by excreta deposition. The previous studies also indicated that clear correlations between N_2_O fluxes and variables of weather conditions were frequently not found under field conditions [41], probably due to the complex interacting influences of different regulators on soil N_2_O emissions under field conditions. In the current study, stronger correlation of N_2_O fluxes with soil temperature than with soil moisture probably implied that soil temperature was a more important regulator for N_2_O emissions at the studied site.

N_2_O fluxes are naturally very varied [39], [44], [45]. This is mainly because soil N_2_O production depends strongly on N- and C-substrate availability, O_2_ concentration, soil temperature and soil moisture, all of which display large temporal and spatial variation, with large emissions observed in wet but not saturated soil with high soil N- and C availabilities [45]. Similar to our study, Saggar et al. [44] reported large variation in ungrazed grassland with coefficient of variation values ranging between 35–59%. Variability increased as a result of animal treading and unevenly distributed excretal returns [41], [44]. For example, coefficient of variation values ranging between 56–262% were found for spatial variation of N_2_O fluxes in a New Zealand dairy-grazed pasture [44].

### Comparison with other studies

To our knowledge, there were few studies comparing N_2_O emissions from grazed grass and grass/white clover pastures in Ireland or in other regions with similar climate conditions. Several studies reported N_2_O emissions from grazed pastures in Ireland but mainly focused on heavily fertilized ryegrass-based pastures [46]–[48]. The annual N_2_O emission was estimated to be 11.6 kg N ha^−1^ from grazed grassland fertilized with 346 kg N ha^−1^ in southern Ireland [48]. Hyde et al. [47] studied N_2_O emissions during two years (Nov 9 2001 to Nov 26 2003) from grazed ryegrass pastures with N application rates of 225 and 390 kg N ha^−1^ yr^−1^, and they found that the emissions were 6.45 and 12.55 kg N ha^−1^ yr^−1^, respectively, in the first year and 18.51 and 28.93 kg N ha^−1^ yr^−1^, respectively, in the second year. Abdalla et al. [46] reported a relative lower level of N_2_O emission (2.4 kg N ha^−1^ yr^−1^) from an extensively grazed ryegrass/white clover pasture fertilized with 200 kg N ha^−1^ yr^−1^. However, their study was conducted on a free-draining soil with a low denitrification potential, and excreta patches were intentionally avoided [46]. Since excreta patches are a major source of N_2_O emission [4], N_2_O emission might have been underestimated when excreta patches were avoided. Reported N_2_O emissions from soils under clover/grass pasture grazed by dairy cows in New Zealand and Australia ranged from 6 to 12 kg N ha^−1^ yr^−1^ [13], [44]. From the comparison, it was concluded that N_2_O emissions from the grazed paddocks in the present study were quite within the reported range.

There were limited data of background N_2_O emissions (N_2_O_Bk_) from grasslands available. The averaged N_2_O_Bk_ from mown grasslands over two years were 0.8 and 1.1 kg N ha^−1^ yr^−1^ for clay and sand sites, respectively, in Netherlands [28]. Flechard et al. [49] reported that N_2_O_Bk_ from the grassland sites involved in EU-GREENGRASS project ranged from −0.5 to 1.2 kg N ha^−1^ yr^−1^. N_2_O_Bk_ ranged from 0.2 to 0.9 kg N ha^−1^ yr^−1^ for grass-clover mixture swards from studies compiled by Rochette & Janzen [24]. N_2_O_Bk_ was 1.0 kg N ha^−1^ yr^−1^ from a ryegrass-white clover sward with a sandy loam texture in Ireland [46]. Substantially higher N_2_O_Bk_ (4.21 and 4.66 kg N ha^−1^ yr^−1^ in 2002 and 2003, respectively) was reported in grassland with a clay loam texture in Ireland [47]. It seemed that N_2_O_Bk_ observed in the current study was relatively high although within the reported range. The relatively high N_2_O_Bk_ for Irish pastures merits further investigation.

### Comparison of the measured and simulated values

Although DNDC was used to simulate N_2_O emissions in the present study, it is important to compare the measured and simulated data of plant growth, soil temperature and moisture. Plant growth plays an important role in regulating the soil water and N regimes, which could further affect a series of biochemical or geochemical processes occurring in the soil. Soil moisture and temperature are two important regulators for soil N_2_O production [50]. The accuracy of the N_2_O emission prediction by models strongly depends on the accuracy of the simulation of soil water status, temperature and mineral N (NH_4_^+^ and NO_3_^−^) contents in the topsoil [51]. Although daily herbage production data were not available, annual production was measured to be 8510 and 7762 kg DM ha^−1^ yr^−1^ for GWC+FN and GWC-FN, respectively, for year 2009 in the adjacent paddocks of the same treatment at the Solohead Research Farm [29]. The simulated and measured herbage DM was quite similar, with RB of 9.1% and 0.67%, respectively. It was clear that DNDC performed very well for the simulation of plant growth, soil temperature and moisture in the present study.

Compared with the simulated data, some peaks of N_2_O fluxes were missed in the measurements probably due to the low sampling intensity. For example, there was a high flux peak for all the treatments in the simulated data around 25 April, 2010 (starting from 24 and ending on 26 April) which was missed in the field measurement. Since there was no rainfall 17 days prior to 24 April, the high N_2_O emissions were like caused by ‘pulsing’ emission, which was typical shortly following rainfall after an extended dry period [42]. However, the peak fluxes for GG+FN and GWC+FN were substantially higher than for other treatments. Since urea was applied to GG+FN and GWC+FN on April 10, the high peaks for these two treatments were likely an accumulation of both pulsing emissions from soil rewetting and fertilization with the latter being dominant. Similarly, Williams et al. [41] reported that peak emissions from fertilization was delayed due to lack of rainfall following fertilization. During the grazing period, the measured fluxes and the simulated values generally matched well for the three grazing treatments, but some simulated and measured peaks did not fit well or some simulated peaks were not found in the measurements due to the low sampling intensity. Similar results were reported by some previous studies simulating N_2_O emissions from grazed pastures [44], [52].

DNDC has been widely validated and used to simulate N_2_O emissions from agricultural soils covering various climate conditions [33], but only a few studies focused on grasslands [37], [44], [53], [54], and few simulated N_2_O emissions from intensively grazed pastures [44], [52], [55]. The existing studies which simulated N_2_O emissions from intensively grazed pastures were mostly carried out in New Zealand. The modified NZ-DNDC (the New Zealand version of DNDC) was very well able to predict the annual measured N_2_O emissions from both the grazed and ungrazed grasslands [44], [52]. Similar to our study, Saggar et al. [52] reported that although the NZ-DNDC generally matched the data, on certain days it tended to over- or underestimate the mean fluxes. A possible explanation for these discrepancies may be the result of very high natural spatial variability in fluxes caused by the heterogeneities in the spatial distribution of excretal N [52]. Theoretically, the large spatial heterogeneity of N_2_O emission requires extensive chamber coverage, while large temporal variation requires a higher sampling frequency. It was observed that sampling at 3- to 7-d intervals resulted in a spread of deviations that ranged from −18 to +24% of the ‘true’ cumulative N_2_O-N emissions (emissions obtained from automated chambers with a sampling intensity of 6 h), and that sampling at 14 d intervals resulted in cumulative estimates that ranged from −43 to 64% of the ‘true’ cumulative N_2_O-N emissions [56]. For the present study, the fact that there were only limited sampling days and chambers undoubtedly contributed to the discrepancy in simulated and measured fluxes. In addition, despite overall correlation, some discrepancies between measured and simulated WFPS occurred (Figure 3), which would lead to further differences between measured and simulated N_2_O emissions.

### Applicability of DNDC to Irish grasslands for N_2_O emission estimate

Both our study and other studies indicated that there was substantial temporal and spatial variation in N_2_O emissions. Hence there are still large uncertainties in the inventory estimate of soil N_2_O emissions despite many years' of measurements. Process-based biogeochemical models, including DNDC, provide the potential to obtain more realistic estimates of soil N_2_O inventory because in appropriate forms they can relate the soil and environmental variables responsible for N_2_O emissions to the size of those emissions [57]. Under the United Nations Framework Convention on Climate Change (UNFCCC), Ireland is required to provide annual greenhouse gas reporting. Currently, Tier 1 method is used to estimate soil N_2_O emissions in the national inventory reporting of Ireland disregarding all site-specific controls and limitations [58]. Ireland is expected to adopt Tier 3 method for soil N_2_O emission estimate by using appropriate models. However, these models should only be used after validation by representative experimental measurements [57].

DNDC has been used to simulate N_2_O emission from intensively [55] and extensively [37] grazed pastures in Ireland. Hsieh et al. [55] found that DNDC well predicted N_2_O emission with a RB of 33% by using site-specific data. However, Abdalla et al. [37] reported RB of 150 and 360% for fertilized and unfertilized plots, and thus they concluded that DNDC was unsuitable for predicting N_2_O from Irish grassland due to its overestimation of WFPS and effect of SOC on the flux [37]. It should be noted that the simulated aboveground DM yield was unreasonably high (33 t ha^−1^) [36]. Since plant growth plays an important role in regulating the soil water and N regimes, which could further affect a series of biochemical or geochemical processes occurring in the soil, the model should be used with caution when simulated and measured plant growth did not fit reasonably well.

In the current study, by inputting the site-specific data, results from DNDC seemed to fit reasonably well with the measured emissions. Considering that there were great uncertainties in the field measurements of N_2_O emissions *per se*, these discrepancies should be acceptable. However, more validation work is needed before DNDC can be formally used for national inventory reporting.

### The contribution of biological N fixation to N_2_O emission and its implications for N_2_O emission mitigation

Although white clover is the main legume in pastures and meadows of temperate regions, there were few field studies comparing N_2_O emissions from grass and grass/white clover grasslands under similar conditions. Ruz-Jerez et al. [59], using acetylene incubation method, estimated N_2_O emissions from New Zealand sheep-grazed grass/clover sward and FN-based grass sward receiving 400 kg N ha^−1^ yr^−1^ and found that both the total N_2_O emissions and the N_2_O production per ton of dry matter produced were higher for the grass sward, despite the higher dry matter production of this system. They further estimated that the N_2_O emissions represented 1.3% of FN and about 1% of biologically fixed N [59]. Šimek et al. [60] reported that N_2_O emissions over a period of 224 days from fertilized ryegrass plots (210 kg N ha^−1^) was 1.4 kg N_2_O-N ha^−1^, much higher than from red clover (0.9 kg N_2_O-N ha^−1^) or grass + red clover plots (0.9 kg N_2_O-N ha^−1^), the latter two treatments receiving a N rate of 20 kg N ha^−1^ in April. In another study, however, annual N_2_O emissions from the grass sward (3.2 and 4.1 kg N ha^−1^) were much lower than from the grass/white clover sward (6.4 and 7.6 kg ha^−1^) despite that the grass sward received 220 kg N ha^−1^ as NH_4_NO_3_ [4]. A laboratory study, where mixtures of white clover and ryegrass were incubated for 14 days in a growth cabinet with a ^15^N_2_-enriched atmosphere, indicated that only 2.1% of the total emitted N_2_O–N originated from recently fixed N implying that recently fixed N released via easily degradable clover residues appeared to be a minor source of N_2_O [22].

In the present study, G-B and WC-B did not receive N fertilization and the comparison of N_2_O emissions between the two treatments could provide some information of the role of BNF in N_2_O emission. The common potential processes responsible for N_2_O production in both treatments were: (i) nitrification following the mineralization of SOM; (ii) denitrification following nitrification; (iii) other processes like nitrifier denitrification which reduces NO_2_^−^ to N_2_ via N_2_O [6]. But for WC-B another possible N_2_O source was BNF itself if there was any. Mineralization of SOM and atmospheric N deposition were major N sources for both G-B and WC-B. But mineralization of residual biologically fixed N was another major N source for WC-B. Biologically fixed N in GWC-FN was 100 and 133 kg N ha^−1^ in 2008 and 2009, respectively [29]. The fixed N in WC-B should be higher than GWC-FN since N fertilization was found to decrease BNF [61]. These fixed N in clover residue provided an important N source for N_2_O production. However, in the current study there was only slight difference between annual N_2_O emissions from G-B and WC-B. This may be a result of higher N use efficiency for WC-B due to grass-legume interactions and efficient transformation of N into biomass [17]. Although the contribution of BNF itself and N input from clover residual decomposition to total N_2_O emission was not quantified in the current study, our data demonstrated that N_2_O emission from BNF itself and white clover residual decomposition in permanent ryegrass/white clover was negligible, which justified the current IPCC methodology in calculating N_2_O emissions attributed to BNF for permanent pastures, i.e., (i) BNF is removed as a direct source of N_2_O because of the lack of evidence of significant emissions arising from the fixation process itself, and (ii) the N in crop residue (including N-fixing crops) for perennial forage crops is only accounted for during periodic pasture renewal [57].

However, the effects of fixed N should be accounted for in grazed clover/grass pastures *via* N2O emissions from excreta (derived from consumed clover) and from increased grass growth (which is consumed and excreted) from mineralized clover N residues, since the conversion of consumed N into product is low and a substantial amount of N (>70%) is recycled through the direct deposition of animal excreta [13]. As indicated in the current study, the peak caused by excreta patch in October accounted for about one third of the annual emission for GWC-FN. However, based on the current study, the contribution of excreta could not be further quantified.

A study conducted during 2004 to 2006 indicated that with the same stocking density (2.2 cows ha^−1^) there was no statistical difference in milk production between GG+FN and GWC+FN (14.3 ton ha^−1^ yr^−1^ for both systems) with concentrate supply of 541 and 559 kg DM cow^−1^ for GG+FN and GWC+FN, respectively [15]. Another study conducted in 2008/2009 revealed that milk production was much higher for GWC+FN (13.7 ton ha^−1^ yr^−1^) than for GWC-FN (10.4 ton ha^−1^ yr^−1^) [30]. These data in combination with N_2_O emissions measured in the present study indicated that there were 0.55 and 0.44 kg N_2_O-N loss per ton of milk production for GG+FN and GWC+FN, respectively, based on 2004–2006 data, or 0.46 and 0.63 kg N_2_O-N loss per ton of milk production for GWC+FN and GWC-FN, respectively, based on 2008/2009 data. This indicated that GWC+FN system was the most efficient in lowering N_2_O emission when economic considerations were taken into account.

### Conclusions

Our study indicated that N_2_O emissions due to biological N fixation itself and clover residual decomposition from permanent ryegrass/white clover grassland were negligible, which confirmed the exclusion of biological N fixation as a direct source of N_2_O emission from the IPCC methodology. Annual N_2_O emissions from the two clover based systems were much lower than from the fertilized system. The process-based DNDC model simulated N_2_O fluxes reasonably well when compared with the measured values. When economic consideration was taken into account, the GWC+FN system should be recommended. Our results indicated that soil N_2_O emissions could be greatly lowered when the GG+FN system was replaced by the GWC+FN system.
